# Acute Inhibition of PI3K-PDK1-Akt Pathway Potentiates Insulin Secretion through Upregulation of Newcomer Granule Fusions in Pancreatic β-Cells

**DOI:** 10.1371/journal.pone.0047381

**Published:** 2012-10-15

**Authors:** Kyota Aoyagi, Mica Ohara-Imaizumi, Chiyono Nishiwaki, Yoko Nakamichi, Kohjiro Ueki, Takashi Kadowaki, Shinya Nagamatsu

**Affiliations:** 1 Department of Biochemistry, Kyorin University School of Medicine, Tokyo, Japan; 2 Department of Diabetes and Metabolic Diseases, Graduate School of Medicine, University of Tokyo, Tokyo, Japan; University of Colorado Denver, United States of America

## Abstract

In glucose-induced insulin secretion from pancreatic β-cells, a population of insulin granules fuses with the plasma membrane without the typical docking process (newcomer granule fusions), however, its mechanism is unclear. In this study, we investigated the PI3K signaling pathways involved in the upregulation of newcomer granule fusions. Acute treatment with the class IA-selective PI3K inhibitors, PIK-75 and PI-103, enhanced the glucose-induced insulin secretion. Total internal reflection fluorescent microscopy revealed that the PI3K inhibitors increased the fusion events from newcomer granules. We developed a new system for transfection into pancreatic islets and demonstrated the usefulness of this system in order for evaluating the effect of transfected genes on the glucose-induced secretion in primary cultured pancreatic islets. Using this transfection system together with a series of constitutive active mutants, we showed that the PI3K-3-phosphoinositide dependent kinase-1 (PDK1)-Akt pathway mediated the potentiation of insulin secretion. The Akt inhibitor also enhanced the glucose-induced insulin secretion in parallel with the upregulation of newcomer granule fusions, probably via increased motility of intracellular insulin granules. These data suggest that the PI3K-PDK1-Akt pathway plays a significant role in newcomer granule fusions, probably through an alteration of the dynamics of the intracellular insulin granules.

## Introduction

Insulin secretion from pancreatic β-cells shows a characteristic biphasic pattern consisting of a rapidly developing and transient first phase followed by a sustained second phase [1]. Observation of the dynamic behavior of insulin granules in glucose-stimulated β-cells by total internal reflection fluorescent (TIRF) microscopy revealed that the exocytosis of insulin granules could be classified into two groups based on distinct behaviors prior to fusion; “previously docked granules” that docked on the plasma membrane before the onset of stimulation, and “newcomer granules” that were originally stored intracellularly [2], [3]. Our previous studies demonstrated that the molecular mechanisms involved in these two types of fusion events were different. The glucose-induced fusion events from previously docked granules were dependent on syntaxin1A and sensitive to the reduction of VAMP2 and SNAP-25 by interleukin-1β (IL-1β) treatment. However, newcomer granule fusions were detectable in pancreatic β-cells genetically lacking syntaxin1A and those treated with IL-1β [3], [4]. Thus, in this study, we aimed to elucidate the molecular mechanism of the glucose-induced insulin secretion, in particular the mechanisms underlying the regulation of newcomer granule fusions.

Recently, we found that treatment with a class IA-selective phosphoinositide 3-kinase (PI3K) inhibitor markedly increased the number of fusion events from newcomer granules [5]. The class IA PI3K preferentially phosphorylates phosphatidylinositol-4,5-bisphosphate (PIP_2_) to generate phosphatidylinositol-3,4,5-triphosphate (PIP_3_), and several molecules bind to PIP_3_ to mediate signals to downstream pathways [6], [7]. In other cell types, PI3K was reported to regulate the trafficking of intracellular vesicles and exocytosis [8], [9]. Particularly, the PI3K-activated Rac1-GEF dependent Rac1 pathway [10], Arf6-GEF dependent Arf6 pathway [11] and 3-phosphoinositide dependent kinase-1 (PDK1)-Akt pathway [12] were demonstrated to regulate vesicular insertion into the plasma membrane and hormone secretion. Thus it is reasonable to assume that PI3K and its downstream effector(s) would be involved in the regulation of the dynamics and exocytosis of newcomer granules in pancreatic β-cells. It is widely known that classical PI3K inhibitors, wortmannin and LY294002, potentiate glucose-induced insulin secretion in mouse and rat islets [13], [14] and β-cell derived Min6 cells [15], but the mechanism is unknown. Therefore, to elucidate the molecular mechanism underlying the regulation of newcomer granule fusions by PI3K, it was necessary to identify the downstream pathway involved in the PI3K inhibitor-induced upregulation of newcomer granule fusions.

In the present study, we investigated PI3K downstream effectors to identify the signaling pathway regulating newcomer granule fusions using a series of constitutive active mutants together with pharmacological inhibitors in pancreatic islets and β-cells. Although virus-mediated gene transfer is widely used in pancreatic islets and β-cells, not all but only a subpopulation of β-cells can be infected. To circumvent this problem, we established a new transfection system which enables us to co-transfect human growth hormone as a reporter, and thereby precisely assess the effect of constitutive active mutants on the glucose-induced secretion. Our results indicated that the acute inhibition of PI3K-PDK1-Akt pathway activated the motility of intracellular insulin granules and increased the glucose-induced insulin secretion by the upregulation of newcomer granule fusions.

## Materials and Methods

### Plasmids

cDNA fragments encoding the full open reading frames of mouse PDK1, Akt1 and PKCζ were amplified from total RNA isolated from Min6 cells and mouse cerebrum by RT-PCR. The purified PCR products were cloned into pENTR1A vector (Invitrogen, Carlsbad, CA). A point mutation of PDK1 to generate constitutive active PDK1 (PDK1(A280V)) [16] was introduced by QuikChange Mutagenesis kit (Agilent Technologies, Santa Clara, CA). A constitutive active Akt1 (myr-Akt1) was generated by adding the Src myristoylation sequence at the N-terminus of Akt1 by PCR [17]. A truncated form of PKCζ lacking the psudosubstrate domain was constructed to generate constitutive active PCKζ (PKCζ(ΔPS)) by PCR [18]. The constitutive active mutant of ARF6 (ARF6(Q67L)), a gift from Dr. M. Takahashi (Kitasato University School of Medicine, Japan) [19], the constitutive active mutant of Rac1 (Rac1(V12)), generously provided from Dr. Y. Takai (Kobe University Graduate School of Medicine, Japan) [20], were subcloned into the pENTR1A vector. To generate the mammalian expression vectors for PDK1(A280V), myr-Akt1, PKCζ(ΔPS), ARF6(Q67L) and Rac1(V12), these sequences were introduced into the pcDNA3.2/V5-DEST vector (Invitrogen) by site-specific recombination according to the manufacturer’s instructions. All constructs were sequenced to verify the identity with a designed sequence.

### Pancreatic Islets and β-cells

Pancreatic islets of Langerhans were isolated from male C57BL/6 mice or male p85α/p85β double knockout mice [21] by collagenase digestion, as described previously [3]. To assay the insulin release assay from islets, isolated islets were washed twice with culture medium then cultured on 60 mm culture dishes (Iwaki, Japan) in RPMI 1640 medium (Invitrogen) supplemented with 10% FBS (Invitrogen), 200 U/ml penicillin, and 200 µg/ml streptomycin at 37°C in an atmosphere of 5% CO_2_. For TIRF and Fura-2 fluorometry analysis, the isolated islets were dispersed and cultured on fibronectin-coated high refractive index coverslips (Olympus, Japan) and normal coverslips (Matsunami, Japan), respectively [5]. Animal experiments were approved by the Kyorin University Animal Care Committee (Permission number: 65-3).

### Insulin Release Assay from Isolated Islets

All insulin secretion experiments were performed at 37°C in Krebs-Ringer buffer (KRB) containing 110 mM NaCl, 4.4 mM KCl, 1.45 mM KH_2_PO_4_, 1.2 mM MgSO_4_, 2.3 mM calcium gluconate, 4.8 mM NaHCO_3_, 2.2 mM glucose, 10 mM HEPES, pH7.4 and 0.3% bovine serum albumin. For the static incubation assay, 10 size-matched islets were preincubated for 30 min in KRB containing 2.2 mM glucose, then transferred into 1 ml of KRB containing 2.2 mM or 16 mM glucose. After 30 min incubation, 200 µl of the supernatant were recovered as the secreted insulin sample. At the end of the experiments, islets were solubilized by 1% Triton-X100 and sonicated on ice. For the perifusion assay, 40 size-matched islets were housed in a small chamber and perifused with KRB containing 2.8 mM glucose for 30 min at a flow rate of 0.5 ml/min. Insulin release was stimulated by 22 mM glucose for 30 min. After the experiments, islets were solubilized by 1% Triton X-100 to recover the total cellular content of insulin. The secreted insulin and total cellular content were measured by an insulin ELISA kit (Morinaga, Japan).

### Islet Transfection and hGH Release Assay

Freshly isolated islets were suspended in 0.5% BSA-containing Hank’s balanced salt solution (Invitrogen) with 1.0 or 1.5 mg/ml DNA and placed in an electrode chamber. Six rectangular electric pulses (200 V, 1-ms duration, 50-ms interval, decay constant: 10%) followed by five pulses (50 V, 50-ms duration, 50-ms interval, decay constant: 40%) and another five pulses in the opposite direction of the electric field were delivered using a pulse generator (Nepa21, Nepa gene). The electroporated islets were washed twice with culture medium and plated on 35 mm or 60 mm culture dishes. Two days after transfection, 15 size-matched islets were subjected to hGH release assay as described above. The secreted hGH and total cellular content of hGH were measured by a hGH ELISA kit (Roche, Applied Science, Indianapolis, IN).

### TIRF Microscopy

The Olympus total internal reflection system with a high aperture objective lens was used as described previously [5]. Briefly, primary cultured β-cells expressing insulin-GFP on the coverslip were mounted on an open chamber and incubated with KRB containing 2.2 mM glucose for 30 min. Stimulation with glucose was achieved by the addition of KRB containing 52 mM glucose into the chamber for a final concentration of 22 mM glucose. Data analysis was perfomed using Metamorph software. Fusion events were manually counted while looping 3500 frame time-lapse. The motions of insulin granules were manually tracked using Metamorph software, and the mean square displacement was determined as reported previously [22]. Quantified data are represented as mean ± S.E.M.

### Immunostaining of Transfected Cells

After transfection of freshly isolated islets with hGH-venus as described above, islets were dispersed and cultured on fibronectin-coated coverslips. After 2 days, cells were fixed and made permeable with 4% paraformaldehyde/0.1% Triton X-100 and were processed for immunostaining as described previously [5]. Cells were labeled with mouse anti-insulin antibody (Sigma, St Louis, MO) and processed with goat anti-mouse IgG conjugated with Alexa Fluor 546 (Invitrogen). Immunofluorescence was detected by a laser-scanning confocal system (FV1000; Olympus).

### Fura-2 Fluorometry

Fura-2 acetoxymethyl ester (Fura-2 AM; Invitrogen) was loaded into primary cultured β-cells and the ARGUS/HiSCA system (Hamamatsu photonics, Japan) was used for measurements, as described previously [5].

### Immunoblotting

Immunoblotting was performed as described previously [5]. Antibodies against Pi-Akt, Pan-Akt, Pi-GSK3β (Cell signaling technology, Danvers, MA) and α-tubulin (Sigma) were purchased from commercial sources.

## Results

### Acute Inhibition of PI3K Enhances the Glucose-induced Insulin Secretion and Acute and Chronic Inhibition of PI3K has Opposite Effects on the Glucose-induced Insulin Secretion

**Figure 1:**
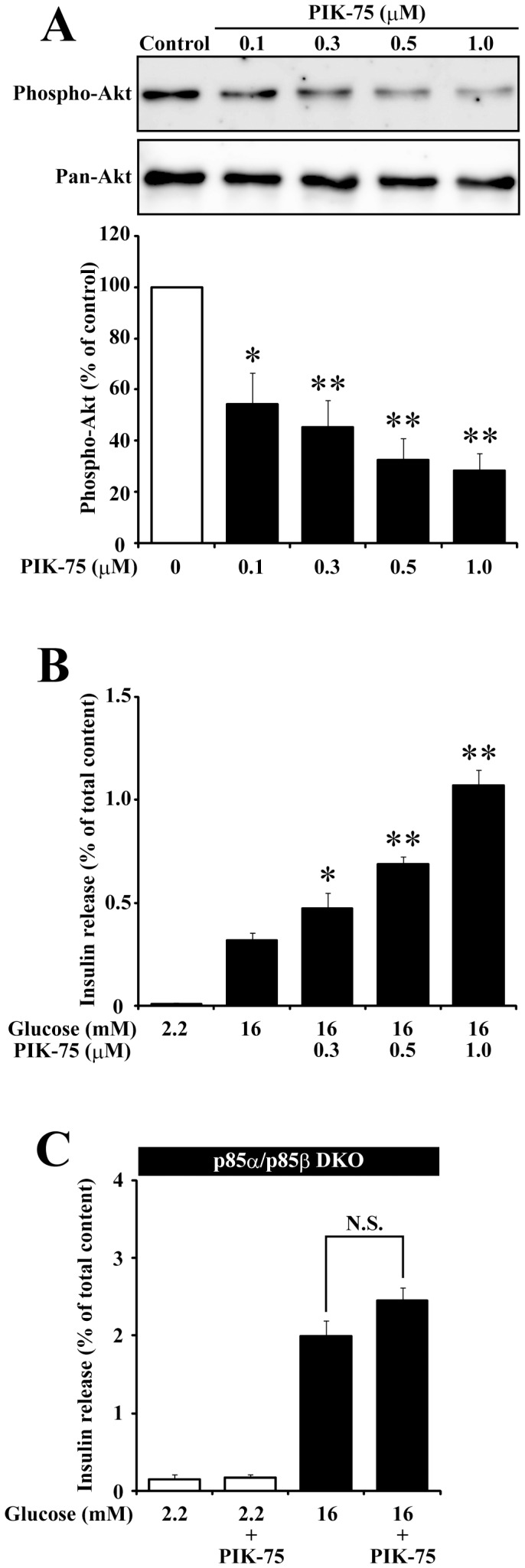
PIK-75 inhibited class IA PI3K and enhanced the glucose-induced insulin secretion. (A) Freshly isolated islets treated with PIK-75 at the indicated concentrations for 30 min were subjected to immunoblotting using anti-Phospho-Akt and anti-Pan-Akt antibodies. Phospho-Akt signal intensity normalized to control was quantified (n = 3 for each group). (B) Freshly isolated islets pre-treated with PIK-75 at the indicated concentrations for 30 min were stimulated with 2.2 mM or 16 mM glucose for 30 min. The amount of secreted insulin were expressed as a percentage of the total cellular content (n = 6 for each group). (C) Pancreatic islets freshly isolated from p85α/p85β DKO mice were pre-treated with or without 0.5 µM PIK-75 for 30 min and stimulated with 2.2 mM or 16 mM glucose for 30 min. The amount of secreted insulin were expressed as a percentage of the total cellular content (n = 6 for each group). Data are the means ± S.E.M. (*, p<0.03; **, p<0.01).

Several previous studies reported that wortmannin enhanced glucose-induced insulin secretion in pancreatic islets and β-cells [13]–[15], but these results are in conflict with a recent report using p85α/p85β double knockout mice in which insulin secretion was impaired [21]. A possible explanation for this discrepancy would be an off-target effect of the classical PI3K inhibitor. To circumvent this possibility, we examined the effect of recently developed PI3K inhibitors (PIK-75 and PI-103) with distinct structures [23] on the glucose-induced insulin secretion in primary cultured mouse islets. First, to examine the effect of PIK-75, freshly isolated islets were incubated with KRB containing PIK-75 at various concentrations for 30 min. As shown in Fig. 1A, PIK-75 greatly reduced the phosphorylation level of Akt. Next, isolated islets were pretrated with PIK-75 for 30 min, then insulin secretion was stimulated by 16 mM glucose. As shown in Fig. 1B, PIK-75 enhanced the glucose-induced insulin secretion in a dose-dependent manner. Another class IA-selective inhibitor PI-103 also dose-dependently suppressed the phosphorylation level of Akt (data not shown) and potentiated the glucose-induced insulin secretion (3.27±0.36% and 7.49±0.36% for control and 0.5 µM PI-103-treated islets, respectively). In order to examine whether the potentiating effect of PIK-75 on insulin secretion would be mediated by specific inhibition of class IA PI3K, islets genetically lacking p85α/p85β was treated with 0.5 µM PIK-75 for 30min then stimulated by 16 mM glucose. We found that PIK-75 was not able to enhance the glucose-induced insulin secretion in p85α/p85β double knockout islets (Fig. 1C). Taken together, these results indicate that the potentiating effect of the class IA-selective PI3K inhibitors on insulin secretion was mediated by the specific inhibition of class IA PI3K.

**Figure 2:**
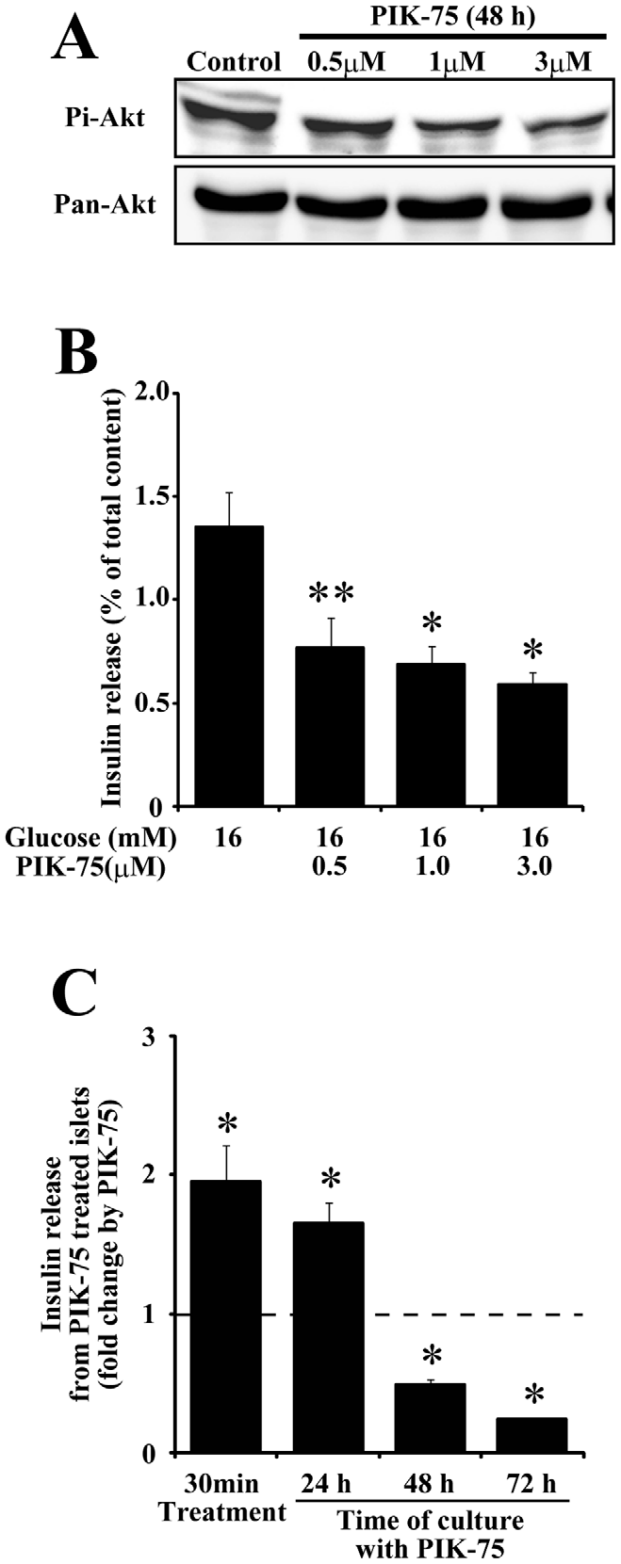
Chronic treatment of isolated islets with PIK-75 reduced the glucose-induced insulin secretion. (A) Pancreatic islets cultured for 48 h with PIK-75 at the indicated concentrations were subjected to immunoblotting using anti-Pi-Akt and Pan-Akt antibodies. (B) Pancreatic islets cultured for 48 h with PIK-75 at the indicated concentrations were stimulated with 16 mM glucose with or without 0.5 µM PIK-75 for 30 min. The amount of secreted insulin was normalized to that from the PIK-75 untreated islets of each group (n = 8 for each group). (C) Freshly isolated islets or those cultured with or without 1 µM PIK-75 for 24, 48 and 72 h were stimulated with 16 mM glucose supplemented with or without 0.5 µM PIK-75 for 30 min. The amount of secreted insulin was normalized to that from the PIK-75 untreated islets of each group (n = 8 for each group). Data are the means ± S.E.M. (*, p<0.01; **, p<0.03).

An alternative possibility for the discrepancy in the results between pharmacological PI3K inhibition and genetic ablation of the PI3K subunit would be the difference in the duration of PI3K inhibition. Thus, we next examined the effect of acute and chronic inhibition of PI3K on the glucose-induced insulin secretion. To suppress the PI3K activity chronically, isolated islets were cultured for 48 h with culture medium supplemented with PIK-75 at various concentrations. The chronic treatment with 0.5 µM PIK-75 reduced the phosphorylation level of Akt by approximately 30%. The phosphorylation level of Akt was further reduced by approximately 40% in islets cultured with 1.0 µM PIK-75 but the chronic treatment with 3.0 µM PIK-75 did not show further reduction of the phosphorylation level of Akt (Fig. 2A). Next, we examined the effect of chronic inhibition of PI3K activity by PIK-75 on the glucose-induced insulin secretion. The glucose-induced insulin secretion was examined in isolated islets cultured with PIK-75 at various concentrations for 48 h. We found that the chronic treatment with PIK-75 significantly suppressed insulin secretion (Fig. 2B). In order to examine the time-dependency of the effect of PIK-75 treatment on the glucose-induced insulin secretion, freshly isolated islets and islets cultured with or without 1 µM PIK-75 for 24–72 h were stimulated with 16 mM glucose. As shown in Fig. 2C, freshly isolated islets treated with PIK-75 for 30 min and those cultured with PIK-75 for 24 h showed significant enhancement of the glucose-induced insulin secretion. On the other hand, when islets were cultured with PIK-75 for 48 h or 72 h, insulin secretion was markedly reduced. Previous reports showed that the classical PI3K inhibitor wortmannin and the genetic ablation of class IA PI3K subunits had the distinct effect on insulin secretion through different mechanisms in terms of gene expression [21], [24]. Consistent with the previous reports using p85α/p85β double knockout mice [21], slight but significant decrease in the expression of syntaxin and SNAP25 was observed in islets cultured with 1.0 µM PIK-75 for 72 h (Fig. S1A). On the other hand, the potentiating effect of PIK-75 on insulin secretion was not affected by the inhibitor of de novo protein synthesis anisomycin (Fig. S1B). These results indicate that the acute PIK-75 treatment potentiates the glucose-induced insulin secretion via specific inhibition of class IA PI3K, whereas the chronic inhibition of PI3K suppresses insulin secretion probably through the downregulation of SNARE protein expression.

**Figure 3:**
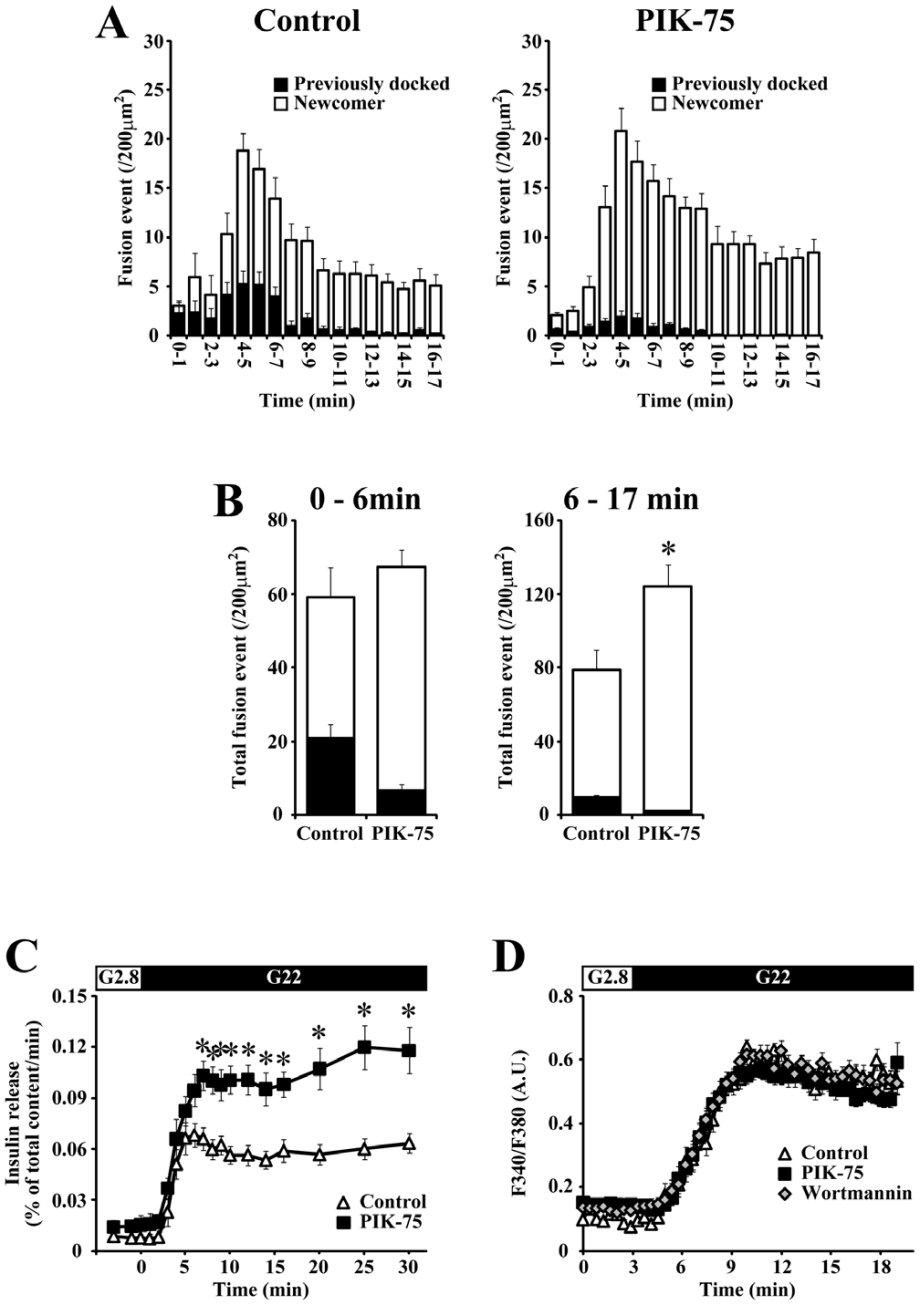
Acute treatment with PIK-75 selectively enhanced the fusions from newcomer granules during the second phase. (A) Pancreatic β-cells expressing insulin-GFP were stimulated with 22 mM glucose at time 0 and the exocytotic responses (events per 200 µm^2^) detected within every 1-min were counted. Histograms show the numbers of fusion events from control (left; n = 10) and 0.5 µM PIK-75-treated cells (right; n = 16). The black column shows fusions from previously docked granules and the white column shows fusions from newcomer granules. (B) Quantitative analysis of the total numbers of fusion events from previously docked (black column) and newcomer (white column) granules detected during the 0–6 min (left) and 6–17 min (right). (C) Control (open triangle; n = 6) and 0.5 µM PIK-75 treated (filled square; n = 6) islets were perifused with 22 mM glucose. (D) [Ca^2+^]_i_ was measured by microfluorometry in Fura-2 loaded β-cells. Control cells (open triangle; n = 31) and cells treated with 0.1 µM wortmannin (open diamond; n = 29) or 0.5 µM PIK-75 (filled square; n = 29) were stimulated with 22 mM glucose at 3 min. The [Ca^2+^]_i_ responses are represented as ratios of the fluorescence intensity. Results are represented as mean ± S.E.M. *; p<0.01.

To examine the effect of the acute inhibition of class IA PI3K on the insulin granule dynamics, we next utilized TIRF microscopy that illuminates a very thin layer immediately adjacent to the coverslips [25] and enables us to analyze the dynamic behavior of insulin granules at a single granule level [2], [3]. Primary cultured β-cells expressing insulin-GFP were incubated with 0.5 µM PIK-75 for 20 min, then the motion of insulin granules was observed under TIRF microscopy. We found that the acute treatment with PIK-75 did not affect the number of docked granules on the plasma membrane (161.7±6.8 and 165.6±10.4 docked granules per 200 µm^2^ for control and PIK-75-treated β-cells, respectively). When β-cells expressing insulin-GFP were stimulated with 22 mM glucose, we detected exocytotic responses that originated from two distinct types of insulin granules with different behaviors prior to fusion: fusions from previously docked and newcomer granules [3]. As shown in Fig. 3A, PIK-75 markedly increased the number of exocytotic responses originating from newcomer granules, whereas fusions that arose from previously docked granules were decreased in PIK-75-treated β-cells. Consistent with the result from the standard perifusion assay (Fig. 3C), a remarkable elevation of the total number of fusion events was detected during the second phase (6–17 min) but not during the first phase (0–6 min), and the upregulation of newcomer granule fusions was entirely responsible for the potentiating effect of PIK-75 on the glucose-induced insulin secretion. (Fig. 3B).

Some reports suggested that classical PI3K inhibitors enhanced the glucose-induced Ca^2+^ influx [26], [27]. However, we observed that neither PIK-75 nor wortmannin affected the [Ca^2+^]_i_ dynamics (Fig. 3D). A previous study showed that wortmannin increased the amount of intracellular cAMP by an unidentified pathway [28]. In our experiments, PIK-75 did not alter the amount of intracellular cAMP (1.16±0.51 and 1.03±0.56 fmol/islet for control and PIK-75-treated islets, respectively). These results demonstrated that the acute inhibition of class IA PI3K increased the number of newcomer granule fusions and potentiated the glucose-induced second phase secretion without affecting [Ca^2+^]_i_ and cAMP, suggesting that a downstream pathway of class IA PI3K would play an important role in the upregulation of newcomer granule fusions.

### Establishment of New System for Transfection into β-cells in Pancreatic Islets

PIK-75 potentiated the second phase insulin secretion in parallel with the upregulation of newcomer granule fusions, and a downstream pathway of class IA PI3K was assumed to mediate the potentiating effect of PIK-75 on insulin secretion. In order to identify the signaling pathway mediating the potentiating effect of PIK-75 on insulin secretion, it was necessary to activate or inhibit specific signaling pathways using constitutive active or dominant negative mutants. Here, we used constitutive active mutants of the PI3K pathway to counteract the effect of PIK-75 on insulin secretion because the expression of dominant negative mutants would chronically suppress a specific pathway and would not be expected to mimic the potentiating effect of acute PIK-75 treatment on insulin secretion.

**Figure 4:**
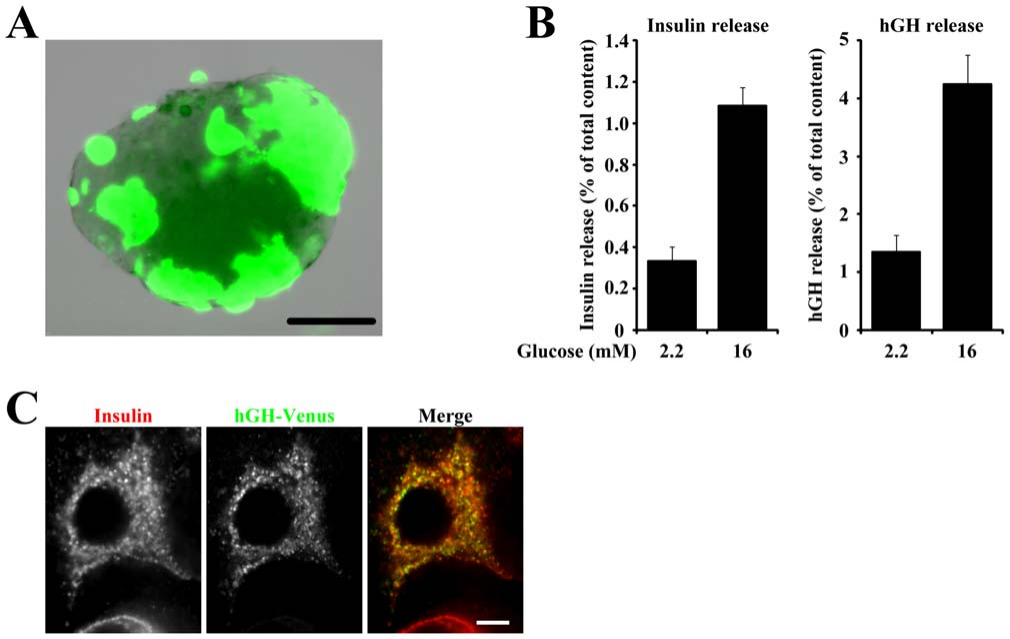
Development of new transfection system. (A) Representative islets transfected with EGFP. Bar = 100 µm. (B) Pancreatic islets transfected with hGH were stimulated with 2.2 or 16 mM glucose, and the amounts of secreted insulin (left) and hGH (right) from the same batch of islets are expressed as a percentage of the total cellular content. Results are means ± S.E.M. (n = 8). (C) Pancreatic islets were transfected with hGH-venus followed by dispersion on coverslips. Two days after transfection, β-cells expressing hGH-venus were immunostained for insulin. Bar = 5 µm.

In pancreatic islets, adenovirus-mediated gene transfer is widely used to cause the expression of exogenous genes and insulin secretion from the infected islets in order to evaluate the effects of the exogenously expressed genes, although not all β-cells in islets can be infected. Therefore, co-transfection of human growth hormone (hGH) as a reporter was required to correctly evaluate the effect of exogenously expressed genes on the glucose-induced secretion specifically in transfected β-cells in islets. Accordingly, we established a new transfection system that enabled us to introduce exogenous plasmids into isolated islets by electroporation. As shown in Fig. 4A, in our new transfection system EGFP was expressed in a substantial population of cells in isolated islets. To examine the suitability of our system to evaluate the secretion from transfected β-cells, isolated islets expressing hGH were stimulated with 2.2 or 16 mM glucose, then insulin and hGH secreted into the extracellular medium from the same batch of transfected islets were measured by ELISA (Fig. 4B). The amount of insulin secreted in response to 16 mM glucose was 3.3-fold higher than that in response to 2.2 mM glucose. In addition, the amount of hGH secreted in response to 16 mM glucose was 3.2-fold higher than that in response to 2.2 mM glucose. Therefore, 16 mM glucose induced comparable amounts of insulin and hGH secretion in transfected islets. Immunostaining of hGH-venus transfected cells for insulin revealed that punctate signals of hGH-venus largely colocalized with insulin (Fig. 4C), indicating that a large part of hGH was stored in insulin granules, as previously reported in β-cell derived clonal cells [29], [30]. It was frequently observed that the percentage of hGH secreted from the small fraction of transfected cells was more than the percentage of insulin secreted from the bulk of the cells. This may suggest that newly assembled secretory granules are preferentially secreted rather than older secretory granules, as reported in other cell types [31]. These data indicate that the amount of secreted hGH induced by 16 mM glucose reflected the ability of secretion in transfected β-cells in islets.

### PDK1 Mediates the Potentiating Effect of PIK-75 on Insulin Secretion

Among the downstream pathways of PI3K, we selected Rac1, Arf6 and PDK1 pathways as candidates in the mediation of the effect of PIK-75 on insulin secretion because of the following reasons. First, all three pathways were reported to link PI3K to exocytosis [10]–[12]. Second, the cytoskeleton was assumed to be important for the regulation of newcomer granule fusion because newcomer granules travel a long distance before fusion with the plasma membrane. Rac1 was shown to mediate the PI3K signal to the regulation of the actin cytoskeleton [32]. Third, the Arf6 pathway regulates phosphatidylinositol-4-phosphate 5-kinase to generate PIP_2_ [33], which has an important role as a priming step in the exocytotic process [34]. PIP_2_ is also known to be a key regulator of the cytoskeleton [35]. Fourth, class IA PI3K regulates various cellular functions mainly through the PDK1 pathway [7]. Therefore, we caused the expression of constitutive active mutants of Arf6, Rac1 and PDK1 together with hGH in isolated islets.

**Figure 5:**
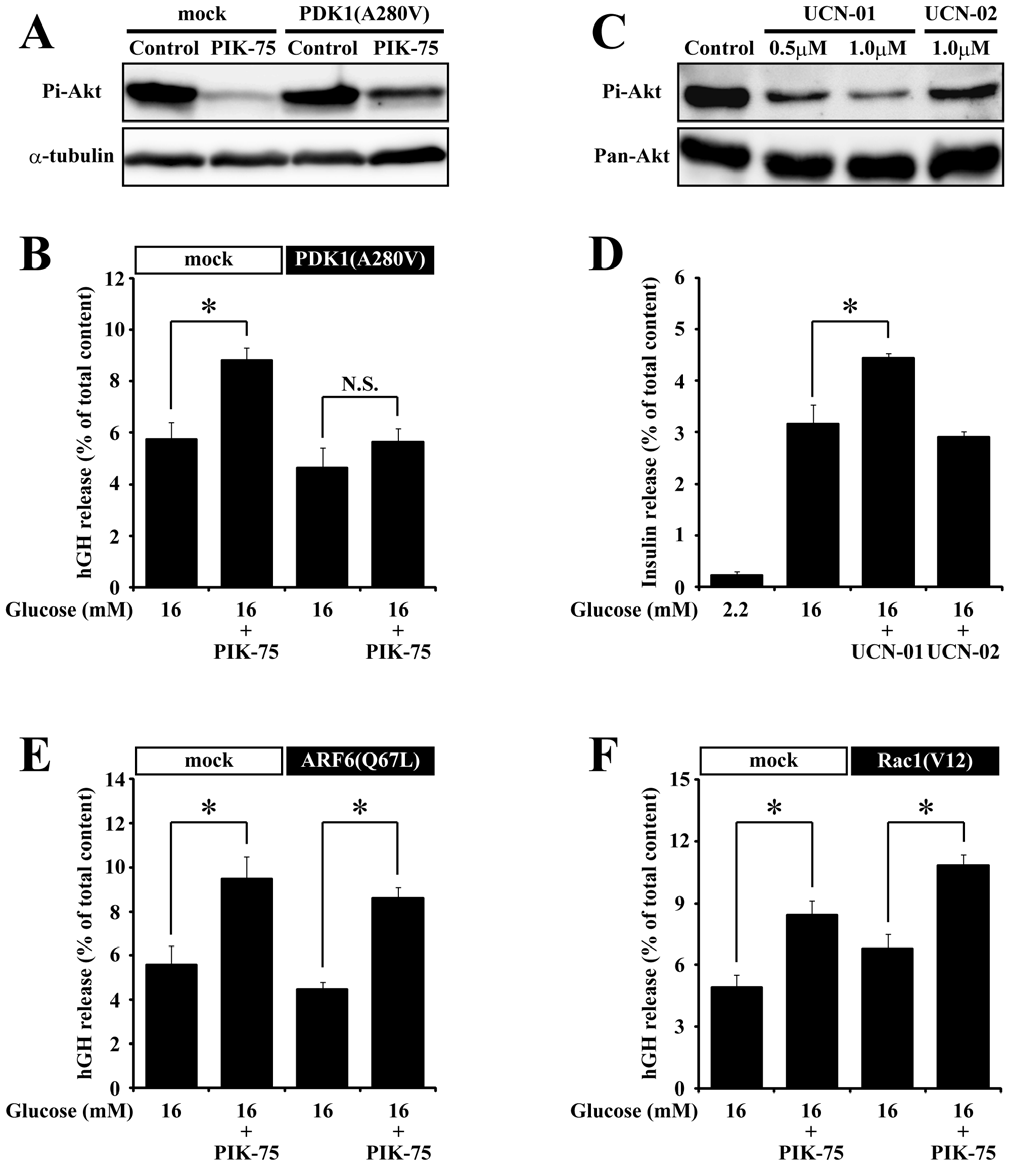
The constitutive active mutant of PDK1counteracted the potentiating effect of PIK-75 on the glucose-induced secretion. (A) COS7 cells transfected with mock or PDK1(A280V) were treated with 0.5 µM PIK-75 for 30 min and subjected to immunoblotting using anti-Pi-Akt and α-tubulin antibodies. Primary cultured islets transfected with PDK1(A280V) (B; n = 6 for each group), Arf6(Q67L) (E; n = 6 for each group) and Rac1(V12) (F; n = 6 for each group) pretreated with or without 0.5 µM PIK-75 for 30 min were stimulated with 16 mM glucose. (C) Cultured islets pretreated with UCN-01 or UCN-02 for 30 min were subjected to immunoblotting using anti-Pi-Akt and Pan-Akt antibodies. (D) Cultured islets pretreated with 1.0 µM UCN-01 or UCN-02 for 30 min were stimulated with 2.2 or 16 mM glucose (n = 8 for each group). Results are represented as mean ± S.E.M. *; p<0.01, N.S.; p>0.3.

The expression of a constitutive active mutant of PDK1, PDK1(A280V) [16], blocked the inhibitory effect of PIK-75 on Akt phosphorylation in COS7 cells (Fig. 5A). The expression of PDK1(A280V) slightly but not significantly reduced the glucose-induced hGH secretion (Fig. 5B). In islets expressing PDK1(A280V), the PIK-75 treatment did not potentiated the glucose-induced hGH secretion, suggesting that PDK1(A280V) suppressed the potentiating effect of PIK-75. Treatment with a PDK1 selective inhibitor, UCN-01, but not with its inactive analogue, UCN-02, inhibited Akt phosphorylation (Fig. 5C) and significantly enhanced the glucose-induced insulin secretion in isolated islets (Fig. 3D). A constitutive active Arf6, Arf6(Q67L) [19], slightly but not significantly reduced the glucose-induced hGH secretion (Fig. 5E). However, the expression of Arf6(Q67L) could not block the potentiating effect of PIK-75 on the glucose-induced hGH secretion because the treatment of islets expressing Arf6(Q67L) with PIK-75 enhanced the hGH secretion. A constitutive active mutant of Rac1, Rac1(V12) [20], enhanced the glucose-induced hGH secretion. However, treatment of Rac1(V12)-transfected islets with PIK-75 further enhanced the glucose-induced hGH secretion, indicating that the expression of Rac1(V12) could not block the potentiating effect of PIK-75 (Fig. 5F). These results indicated that the class IA PI3K-PDK1 pathway, but not the Arf6- and Rac1-dependent pathways, mediated the potentiating effect of PIK-75 on insulin secretion.

### PI3K-PDK1-Akt Pathway is Important for the Potentiating Effect of PIK-75 on Insulin Secretion

**Figure 6:**
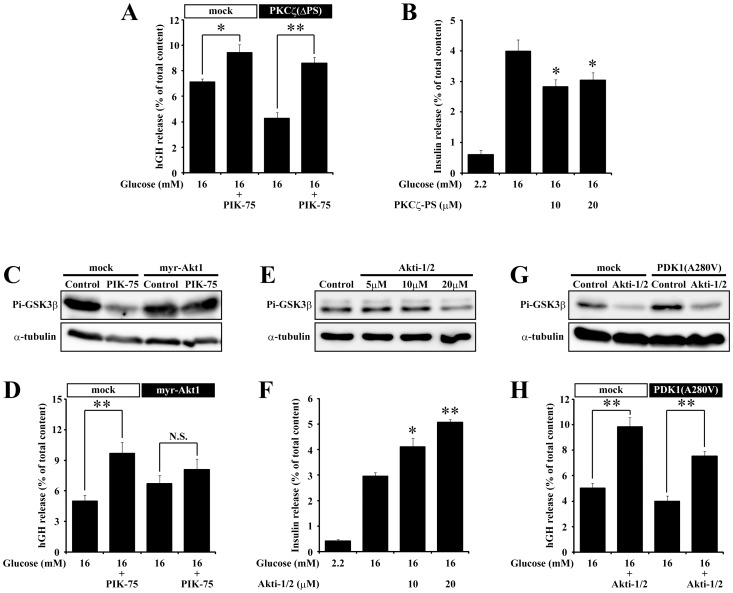
Akt mediated the potentiating effect of PIK-75 on the glucose-induced secretion. Cultured islets transfected with PKCζ(ΔPS) (A; n = 6 for each group), myr-Akt1 (D; n = 6 for each group) and PDK1(A280V) (H; n = 6 for each group) pretreated with or without 0.5 µM PIK-75 or 1 µM Akti-1/2 for 30 min were stimulated with 16 mM glucose. Alternatively, insulin secretion from cultured islets pretreated with PKCζ-PS (B; n = 8 for each group) or Akti-1/2 (F; n = 8 for each group) for 30 min were stimulated with 16 mM glucose. COS7 cells transfected with myr-Akt1 (C) or PDK1(A280V) (G) were treated with or without 0.5 µM PIK-75 or 1 µM Akti-1/2 for 30 min and subjected to immunoblotting using anti-Pi-GSK3β and α-tubulin antibodies. (E) Cultured islets pretreated with Akti-1/2 for 30 min were subjected to immunoblotting using anti-Pi-GSK3β and α-tubulin antibodies. Data are represented as a mean ± S.E.M. *; p<0.03, **; p<0.01; N.S.; p>0.3.

PDK1 phosphorylates and activates atypical PKCs [36], [37] and Akt [38]. To further characterize the signaling pathway involved in the potentiating effect of PIK-75 on insulin secretion, we next examined whether the constitutive active mutants of atypical PKC and Akt could counteract the acute effect of PIK-75 on the glucose-induced secretion. We used a truncated PKCζ as a constitutive active PKCζ, which deleted the psudosubstrate domain (PKCζ(ΔPS)) [18]. As shown in Fig. 6A, the constitutive active PKCζ did not block the potentiating effect of PIK-75 on the glucose-induced hGH secretion. In addition, a specific inhibitor of atypical PKC, myristoylated PKCζ-PS, did not enhance but reduced the glucose-induced insulin secretion (Fig. 6B), suggesting that atypical PKC was not involved in the potentiating effect of PIK-75 on the glucose-induced secretion. On the other hand, the expression of a constitutive active Akt1 mutant, myr-Akt1 [17], suppressed the inhibitory effect of PIK-75 on GSK3β phosphorylation, a downstream target of Akt [39] (Fig. 6C), and blocked the potentiating effect of PIK-75 on the glucose-induced hGH secretion (Fig. 6D). Furthermore, an Akt selective inhibitor, Akti-1/2, reduced the phosphorylation level of GSK3β (Fig. 6E) but potentiated the glucose-induced insulin secretion (Fig. 6F). Because of a possible feedback regulation of PI3K by the Akt pathway [40], we also examined whether PDK1(A280V) might affect the potentiating effect of Akti-1/2 on the glucose-induced secretion. However, the expression of PDK1(A280V) did not affect the potentiating effect of Akti-1/2 on the glucose-induced hGH secretion (Fig. 6H). These results demonstrated that the PI3K-PDK1-Akt signaling pathway was important for the potentiating effect of PIK-75 on the glucose-induced secretion.

### Acute Inhibition of Akt Increases the Motility of the Intracellular Insulin Granules and Enhances the Number of Newcomer Granule Fusions

**Figure 7:**
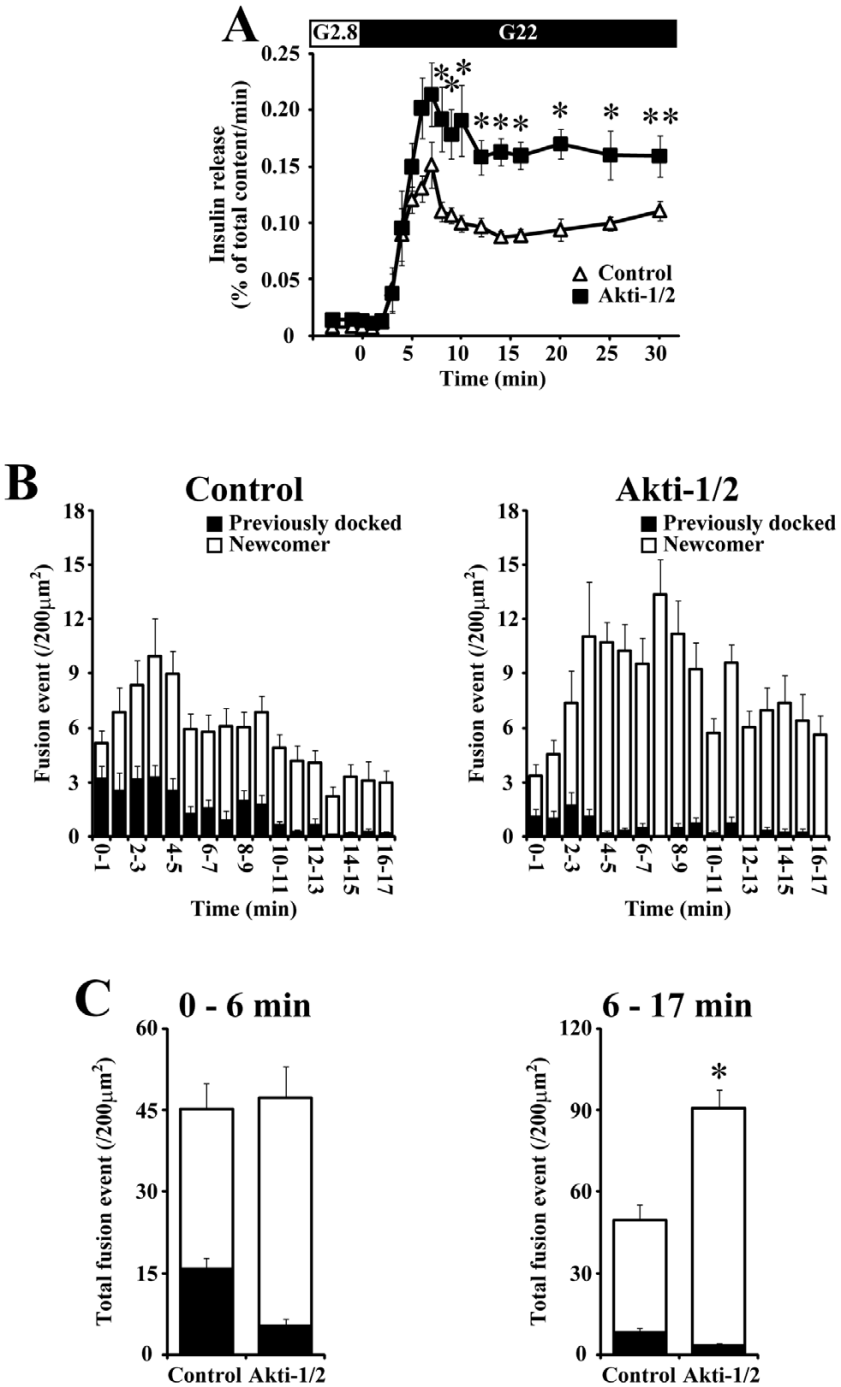
The effect of Akt inhibitor on the glucose-induced insulin secretion. (A) Control (open triangle; n = 6) and 1 µM Akti-1/2 treated (filled square; n = 6) islets were perifused with 22 mM glucose. (B) Pancreatic β-cells expressing insulin-GFP were stimulated with 22 mM glucose and the fusion events (per 200 µm^2^) detected within every 1-min were counted. Histogram showing the numbers of fusion events from control (left; n = 16) and 1 µM Akti-1/2 treated cells (right; n = 10). The black column shows fusions from previously docked granules and the open column shows fusions from newcomer granules. (C) Quantitative analysis of total numbers of exocytotic events from previously docked (black column) and newcomer (white column) granules detected during 0–7 min (left) and 7–16 min (right). Data are represented as mean ± S.E.M. *; p<0.01, **; p<0.03.

From the data described above, the acute inhibition of class IA PI3K and Akt should have the same potentiating effect on the glucose-induced insulin secretion through the upregulation of newcomer granule fusions. Indeed, Akti-1/2 treatment enhanced the glucose-induced insulin secretion mainly during the second phase (Fig. 7A), as we observed using PIK-75 (Fig. 3C). To further evaluate the potentiating effect of Akti-1/2 on the glucose-induced insulin secretion, the dynamic behavior of insulin granules in response to glucose stimulation was assessed by TIRF microscopy. Akti-1/2 did not affect the number of docked granules observed under TIRF microscopy (174.6±7.5 and 175.7±9.2 per 200 µm^2^ for control and Akti-1/2 treated β-cells, respectively). As shown in Fig. 7B and 7C, Akti-1/2 treatment markedly increased the total number of fusion events during the second phase. This was completely achieved by the upregulation of fusions originating from newcomer granules, whereas the number of fusion events that arose from previously docked granules was decreased by the Akti-1/2 treatment. Thus, these phenotypes of the insulin granule dynamics induced by the acute inhibition of Akt were almost equivalent to those induced by PIK-75 treatment (Fig. 3A, B).

**Figure 8:**
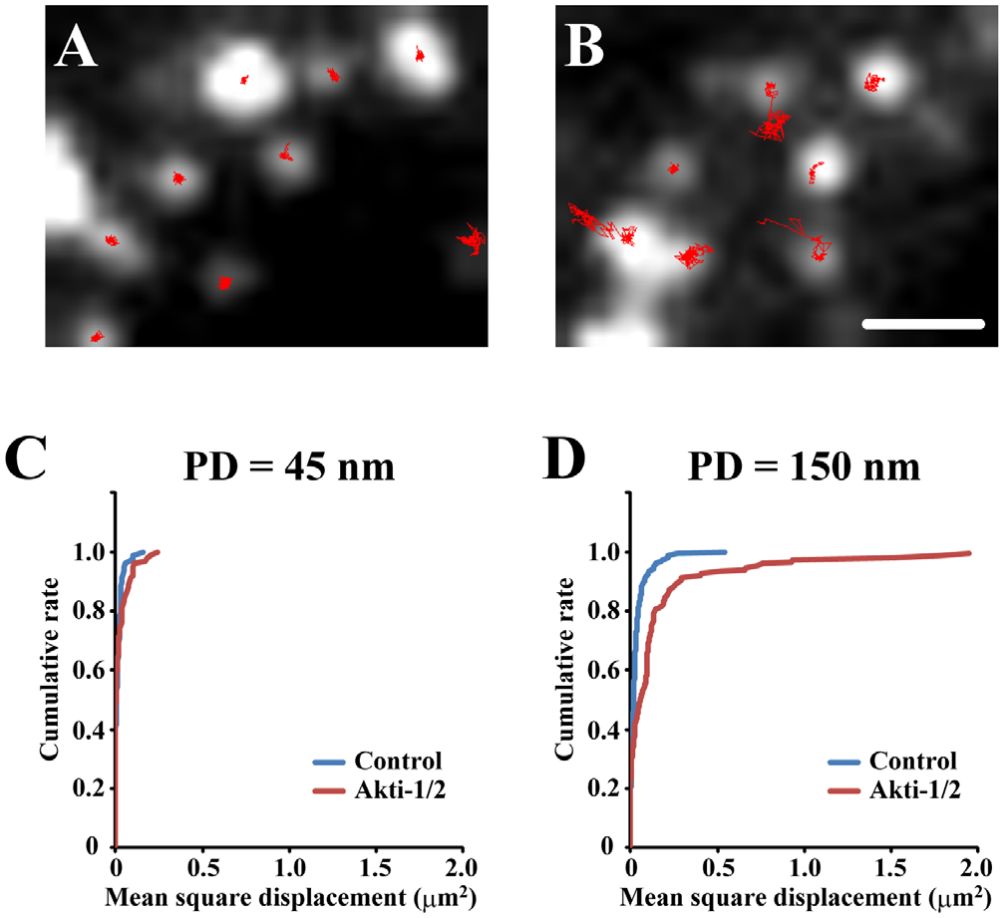
The effect of Akt inhibitor on dynamics of intracellular insulin granules. (A, B) Control (A) and 1 µM Akti-1/2 treated β-cells (B) expressing insulin-GFP were observed under TIRF microscopy with PD = 150 nm and the motions of insulin granules for 1 min were manually tracked (red lines). (C, D) Cumulative plots of mean square displacement for 1min observed in TIRF microscopy with PD = 45 nm (C; n = 79 and 101 for control and Akti-1/2, respectively) and PD = 150 nm (D; n = 154 and 149 for control and Akti-1/2, respectively).

We found that Akti-1/2 treatment increased the motility of the intracellular insulin granules. To analyze the motility of insulin granules adjacent to the plasma membrane and those located intracellularly, we observed β-cells expressing insulin-GFP under TIRF microscopy with a penetration depth = 45 nm and 150 nm. The motion of most insulin granules detected within 100 nm away from the coverslips (penetration depth = 45 nm) was highly restricted as if they were tethered, suggesting that they would molecularly dock on the plasma membrane. On the other hand, a considerable population of insulin granules observed within 345 nm from the coverslips (penetration depth = 150 nm) showed significant lateral movements. To quantify the mobile behaviors, we tracked insulin granules for 1 min and assessed their movement by mean square displacement, as reported previously [22]. As shown in Fig. 8C, the acute inhibition of Akt did not affect the dynamics of docked granules detected under TIRF microscopy. On the other hand, Akti-1/2 treatment induced a marked increase in the proportion of active intracellular insulin granules (Fig. 8D; p<0.01; Kolmogorov-Smirnov test), suggesting that the activation of the insulin granule dynamics by Akti-1/2 would cause the upregulatin of newcomer granule fusions.

## Discussion

In the present study, we found that the enhancement of the glucose-induced insulin secretion induced by PI3K inhibitors was mediated by acute but not chronic inhibition of the PI3K-PDK1-Akt pathway. The inhibition of PI3K-PDK1-Akt pathway selectively enhanced newcomer granule fusions in parallel with the activation of the dynamic behavior of the intracellular insulin granules, suggesting that an effector downstream of Akt would control the number of newcomer granule fusions by regulating the motility of intracellular insulin granules.

We observed that the potentiation of insulin secretion induced by acute inhibition of PI3K-PDK1-Akt was mostly due to the upregulation of newcomer granule fusions (Fig. 5B–C). Because newcomer granules are originally located intracellularly and travel a long distance to the plasma membrane, activation of the dynamic behavior of intracellular insulin granules would be necessary to increase the number of fusion events from newcomer granules. Thus it was reasonable that the increase in newcomer granule fusions induced by Akt inhibition was observed in parallel with the increased motility of intracellular insulin granules (Fig. 8). Therefore, a downstream target of Akt regulating the dynamic behavior of insulin granules should be responsible for the potentiating effect of PI3K inhibitors on insulin secretion. MyosinVa might be a possible candidate for Akt target to regulate newcomer granule fusions. MyosinVa is one of the molecular components of insulin granule trafficking [41] and has been shown to be phosphorylated by Akt [42]. Because the phosphorylated myosinVa by Akt tightly binds to actin cytoskeleton [42], the inhibition of PI3K-PDK1-Akt pathway would detach insulin granules from actin cytoskeleton and might increase the number of insulin granule competent for fusion as newcomer granules.

Although the isoform-nonselective PI3K inhibitors, wortmannin and LY294002, are known to enhance insulin secretion [13]–[15], a recent report using isoform-specific PI3K inhibitors and isoform-specific knockout mice demonstrated that inhibition of class IB and class II PI3K resulted in the reduction of insulin secretion [43]–[45]. Thus, class IA PI3K was a candidate in the mediation the potentiating effect of wortmannin on the glucose-induced insulin secretion. In the present study, we observed that acute treatment of a recently developed class IA-selective PI3K inhibitors enhanced glucose-induced insulin secretion (Fig. 1). We also showed that insulin secretion from islets genetically lacking p85α/p85β was not enhanced by PIK-75 treatment, indicating that the potentiating effect of PIK-75 was mediated by class IA PI3K. Pharmacological inhibitors of class IA PI3K downstream effectors, PDK1 (Fig. 5D) and Akt (Fig. 6F), also potentiated the glucose-induced insulin secretion, and the constitutive active mutant of PDK1 (Fig. 5B) and Akt (Fig. 6D) counteracted the potentiating effect of PIK-75. These results clearly indicated that the acute inhibition of PI3K-PDK1-Akt pathway enhanced the glucose-induced insulin secretion. In contrast to the acute effect of PIK-75, we found that chronic treatment with PIK-75 inhibited insulin secretion (Fig. 2), consistent with the results from the genetic ablation of p85α/p85β [21]. The downregulation of SNARE proteins would cause the impairment of insulin secretion observed in chronic treatment of PIK-75 (Fig. S1) [21]. Although Hashimoto and colleagues demonstrated that PDK1 was indispensable for the regulation of the β-cell mass [46], the role of PDK1 in the glucose induced insulin secretion was not determined. Deletion of Akt2 was shown to increase insulin secretion [47], but the overexpression of the dominant negative form of Akt1 decreased the glucose-induced insulin secretion [48]. Further studies are necessary to reconcile the discrepancy in the effect of long-term inhibition of the Akt pathway on insulin secretion.

Although the PI3K inhibitors potentiate insulin secretion in β-cells, several studies in various cell types showed that the pharmacological inhibition of PI3K resulted in the reduction of exocytosis [9], [49]. In contrast to the enhancement of newcomer granule fusions by acute inhibition of the class IA PI3K-PDK1-Akt pathway, we also observed that the both PIK-75 and Akti-1/2 treatment significantly reduced the exocytotic responses that arose from previously docked granules (Fig. 3B and Fig. 7C). Because the mechanism underlying fusions from previously docked granules in pancreatic β-cells and that in other secretory cells are fundamentally very similar [3], it is reasonable that pharmacological inhibition of PI3K and Akt suppressed the fusions from previously docked granules. In addition, our previous studies demonstrated that the molecular mechanisms involved in fusions from previously docked and newcomer granules were different [3], [4], [50]. Therefore, it would be expected that distinct molecular mechanisms should be exist to mediate Akt signaling to fusions originating from previously docked and newcomer granules. Synip was reported to bind to syntaxin to inhibit ternary SNARE complex, furthermore overexpression of synip in β-cell derived clonal cells resulted in the inhibition of glucose-induced insulin secretion [51]. Phosphorylation of Synip by Akt promotes the dissociation of synip from syntaxin, which allows vesicles to dock and fuse with the plasma membrane [52]. Thus, synip may link the inhibition of Akt activity with the suppression of fusions from previously docked granules. Because the acute inhibition of the class IA PI3K-PDK1-Akt pathway selectively potentiated newcomer granule fusions, further studies to elucidate the mechanism underlying the potentiating effect of PIK-75 and Akti-1/2 on insulin secretion should reveal the molecular mechanism of newcomer granule fusions and biphasic insulin secretion.

In conclusion, the acute inhibition of class IA PI3K potentiated the glucose-induced insulin secretion through the PDK1-Akt dependent pathway in pancreatic β-cells. The potentiation of insulin secretion induced by the acute inhibition of the class IA PI3K-PDK1-Akt pathway was achieved by the increase in the number of exocytotic responses originating from newcomer granules, and the activation of the dynamic behavior of intracellular insulin granules could cause the upregulation of newcomer granule fusions. Further studies are necessary to identify the Akt substrate involved in the potentiating effect of class IA PI3K inhibitors on insulin secretion and elucidate the molecular mechanism underlying newcomer granule fusions.

## Supporting Information

Figure S1 **Effect of chronic and acute PIK-75 treatment on SNARE protein expression.** (A) Pancreatic islets cultured with or without 1.0 µM PIK-75 for 2 days were subjected to immunoblotting using anti-syntaxin, SNAP25 and α-tubulin antibodies. Syntaxin (n = 20 and 11 for control and PIK-75, respectively) and SNAP25 (n = 15 and 8 for control and PIK-75, respectively) signal intensity were normalized to PIK-75 non-treated islets and quantified. (B) Cultured islets treated with or without 40 µM Anisomycin and/or 0.5 µM PIK-75 for 30 min were stimulated with 16 mM glucose for 30 min. The amount of secreted insulin were expressed as a percentage of the total cellular content (n = 6 for each group). Data are the means ± S.E.M. (*, p<0.05; **, p<0.03; ***, p<0.01).
(TIF)
