# Supplementary figures and images for: Acute Inhibition of PI3K-PDK1-Akt Pathway Potentiates Insulin Secretion through Upregulation of Newcomer Granule Fusions in Pancreatic β-Cells
Source: PLoS One. 2012 Oct 15;7(10):e47381. doi: 10.1371/journal.pone.0047381 (PMC3471824; doi:10.1371/journal.pone.0047381)

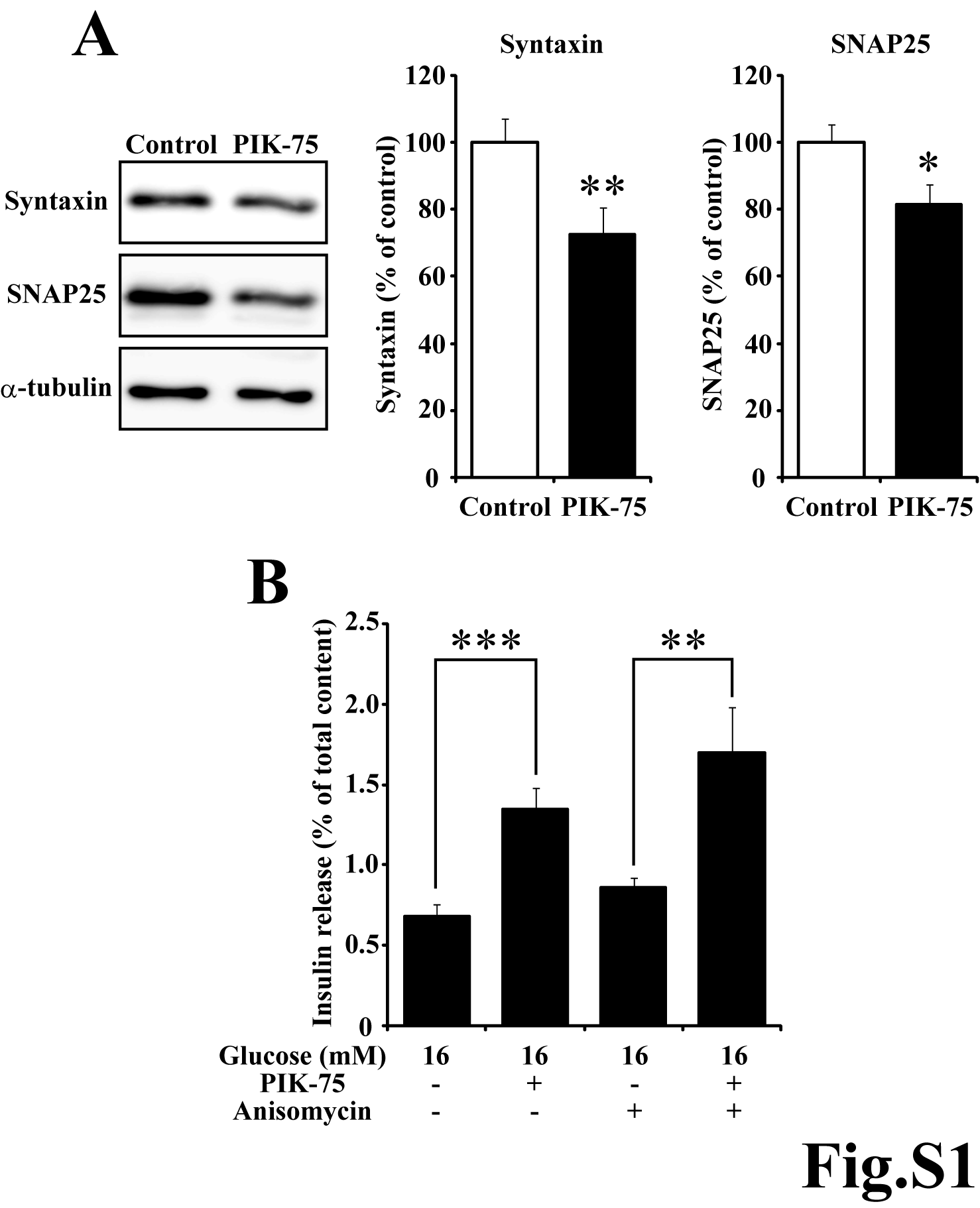

Supplement: Figure S1 — Effect of chronic and acute PIK-75 treatment on SNARE protein expression. (A) Pancreatic islets cultured with or without 1.0 µM PIK-75 for 2 days were subjected to immunoblotting using anti-syntaxin, SNAP25 and α-tubulin antibodies. Syntaxin (n = 20 and 11 for control and PIK-75, respectively) and SNAP25 (n = 15 and 8 for control and PIK-75, respectively) signal intensity were normalized to PIK-75 non-treated islets and quantified. (B) Cultured islets treated with or without 40 µM Anisomycin and/or 0.5 µM PIK-75 for 30 min were stimulated with 16 mM glucose for 30 min. The amount of secreted insulin were expressed as a percentage of the total cellular content (n = 6 for each group). Data are the means ± S.E.M. (*, p<0.05; **, p<0.03; ***, p<0.01). (TIF) [file pone.0047381.s001.tif]
